# Hepatoblastoma Cell Lines: Past, Present and Future

**DOI:** 10.3390/cells14242013

**Published:** 2025-12-17

**Authors:** Edward V. Prochownik, Colin M. Henchy, Huabo Wang

**Affiliations:** 1 Division of Hematology/Oncology, UPMC Children’s Hospital, Pittsburgh, PA 15224, USA; cmh259@pitt.edu (C.M.H.); huw14@pitt.edu (H.W.); 2 Department of Microbiology and Molecular Genetics, School of Medicine, The University of Pittsburgh, Pittsburgh, PA 15213, USA; 3 The Hillman Cancer Center, The University of Pittsburgh Medical Center, Pittsburgh, PA 15232, USA; 4 The Pittsburgh Liver Research Center, The University of Pittsburgh Medical Center, Pittsburgh, PA 15213, USA; 5 Rangos Research Center, Room 5124, 4401 Penn Avenue, Pittsburgh, PA 15224, USA

**Keywords:** β-catenin, *CDKN2A*, *MYC*, NFR2, Wnt, YAP, hepatocellular carcinoma, hepatoblastoma

## Abstract

**Highlights:**

**What are the main findings?**

- Immortalized cell lines can now be routinely established from genetically defined murine hepatoblastomas (HBs).
- In response to hypoxia, some of the cell lines display angiogenic potential that benefits tumor growth.

**What are the implications of the main findings?**

- HB cell lines can be used to test the role of different oncogenic drivers on drug susceptibilities and for the discovery of novel targets.
- The murine HB cell lines have provided guidance for the development of similar cell lines of human origin.

**Abstract:**

Hepatoblastoma (HB), the most common pediatric liver malignancy, tends to be highly curable although advanced or recurrent disease has less favorable outcomes. Because patients are invariably <3–4 years of age, chemotherapies can cause significant long-term morbidities. Immortalized HB cell lines could be of great utility for drug screening, for the identification of novel therapeutic susceptibilities, and for studies requiring highly regulated and/or rapidly changing in vitro environments. However, HB research is hampered by a paucity of these lines that could be used for such purposes, with only two human cell lines being readily available, neither of which represents the most common HB molecular subtypes. Recently, immortalized cell lines have been derived from murine HBs that are driven by the most common oncogenes and tumor suppressors associated with human tumors. These comprise five distinct groups associated with the deregulation of each of the four possible combinations of oncogenic forms of the β-catenin, YAP and NRF2 transcription factors or the over-expression of MYC. All five groups share many of the attributes and molecular signatures of actual human HBs. In addition, they have been used for purposes as diverse as identifying novel molecular targets through the use of Crispr-based screens and the demonstration that some HB cells can trans-differentiate into endothelial cells that facilitate tumor growth. The experience gained from these models and advances in the propagation of human hepatocytes in mice suggests that it may soon be possible to generate bespoke human immortalized human cell lines.

## 1. Introduction

Despite being the most common liver cancer of childhood, hepatoblastoma (HB) remains a rare tumor, even by pediatric standards, with fewer than 100 cases per year occurring in the United States and comprising ~1% of all pediatric cancers world-wide [1]. However, its incidence (~1.8 cases/million) is increasing more rapidly than that of any other pediatric cancer [1]. Fortunately, it is highly curable with current overall survival rates approaching ~90% for lower stage disease following treatment with a combination of surgery and chemotherapy [2,3,4]. In contrast, the success rate declines markedly for more advanced stages (~50–60% survival). Recurrent/refractory disease also tends to be resistant to traditional therapies such that orthotopic liver transplantation is currently considered the treatment of choice for this subset of individuals despite its expense, its limited availability, and its associated subjugation to lifelong immunosuppression and its own long-term morbidities [3,5,6].

Despite the generally positive outcomes associated with current HB treatments, they must be employed judiciously and with the long-term goal of achieving higher cure rates while also reducing side effects. This is particularly important given the above-noted increase in HB’s incidence and the increasing number of individuals who are receiving therapy [1,7]. A better understanding of HB’s underlying pathogenesis and biology is therefore necessary to allow the most specific and least toxic drugs and drug combinations to be identified and tested, particularly in vitro where such studies can be conducted rapidly and efficiently. Although current chemotherapeutic modalities are effective, well-understood and initially well-tolerated, their long-term side effects can be substantial and are at least in part a consequence of the particularly young age of HB patients, the vast majority of whom are <3–4 years [8,9]. For these reasons, new and less toxic forms of therapy, more rapid and efficient ways of drug testing and a better understanding of potential molecular targets are essential.

Although improved chemotherapeutic success and long-term survival have long been associated with certain clinical, histologic and biochemical features of HB and, more recently, with its transcriptional signature, all patients still tend to receive the same drugs [10,11,12,13,14]. This is quite unlike the case for most other cancers—particularly those of adult origin—for which therapies are now increasingly “personalized” as tumors are classified according to their gene expression profiles and the presence of targetable oncogenic driver mutations [15,16,17,18]. For HB, clinical trials of new agents based upon gene expression patterns are hampered by the small number of new cases and the already high cure rates as noted above. Thus, both the testing of novel chemotherapeutic agents and the identification of new targets using unbiased techniques such as Crispr screening are best done in cell lines [19,20,21,22,23,24]. In this review we discuss how, until quite recently, the paucity of readily attainable and well-characterized HB cell lines has impeded drug discovery, slowed the identification of novel and specific susceptibilities and likely stymied us from improving cure rates and reducing long-term morbidities. We also describe how this has restricted our ability to answer basic questions concerning HB biology. We then discuss in some detail the recent derivation of several different types of murine HB cell lines of defined molecular origin and how this has improved our ability to address long-standing questions in the field of HB research while providing valuable new resources with which to pursue drug discovery. The advantages and disadvantages of the various murine cell lines are also discussed. Finally, we speculate as to how the knowledge gained from murine HB cell lines points the way toward the ultimate goal of developing genetically defined human HB cell lines without relying on actual tumors.

## 2. Human HB Cell Lines

### 2.1. Background

The establishment of cell lines from primary cancers has never been easy or reliable despite the primary tumor cells already having cleared key barriers to achieving immortalization and unlimited replication that should have allowed them to thrive in vitro [25]. Many non-mutually exclusive and tissue-specific factors likely account for the inability to make this transition. These include genetic and/or epigenetic backgrounds that are either lacking or are poorly adapted for maintaining viability or proliferation in vitro, the absence of or imbalance among essential nutrients or growth factors provided by surrounding normal tissues, and the loss of 3-dimensional relationships and associated mechanical forces that impact matrix stiffness [26,27,28,29,30]. Given that essentially no progress has been made in establishing human HB cell lines since this topic was last reviewed a decade ago [31], researchers have recently shifted their attention to alternative approaches, namely, the generation of murine cell lines that will be discussed in greater detail below [20,32,33].

### 2.2. The Current Status of Human HB Cell Lines

Fewer than a dozen human HB cell lines have been developed, with the vast majority having been neither well-characterized, widely reported nor made available to the wider research community (Table 1). Indeed, a survey of several major commercial vendors of mammalian cell lines (Accegen, Applied Biological Materials, ATCC, Biohippo, Cellosaurus, The Corriell Institute, Cytion, and LifeLine Cell Technology) shows only the HepG2 and Huh6 cells to be obtainable. A number of other cell lines that have been reported such as H7D7A and DPX2 are clonal derivatives of HepG2 cells (https://www.cellosaurus.org/CVCL_1T06 (accessed on 1 November 2025), https://www.cellosaurus.org/CVCL_UK01 (accessed on 1 November 2025)). Thus only eight of these HB cell lines truly derive from independent tumors. Moreover, a PubMed search conducted in Aug 2025 using the names of the human HB cell lines shown in Table 1, with or without the term “hepatoblastoma”, revealed over 43,000 references for HepG2 cells, 250 references for Huh6 cells and fewer than 60 references for all the other cell lines combined. Thus, >99% of studies employing HB cell lines have been performed with the same cell line (HepG2) or its clonal derivatives.

### 2.3. HepG2 Cells

HepG2 cells were originally established from a liver tumor that was mis-identified as a well-differentiated hepatocellular carcinoma (HCC) [39,48]. This incorrect diagnosis may have been partly informed by the patient’s age (15 years), which was much older than that of the typical child with HB, even though HB is occasionally seen in older children and even adults of considerable age [48,49]. The initial confusion was compounded by a subsequent genomic analysis of the cell line that reported nearly 100 mutant forms of various oncogenes and tumor suppressors (TSs), none of which involved the genes that are commonly associated with HB [50,51]. More recent re-examination of the original histologic sections of this tumor along with molecular profiling and comparative genomic hybridization led to the conclusion that HepG2 cells did in fact originate from a classical epithelial-type HB [34,35]. This cell line has subsequently undergone numerous in vivo and in vitro selections, adaptations and sub-clonings and has likely experienced considerable genetic drift although formal comparisons among the different sub-lines are lacking. It is also highly aneuploid and carries numerous trisomies, translocations and deletions [52]. Attesting to the notion that considerable genetic drift has occurred is the fact that independent studies have described different sizes of in-frame deletions involving exons 3 and 4 of the *CTNNB1* gene that encodes β-catenin (or CTNNB1), the major oncogenic driver of HB [10,36,37,38,52,53,54,55,56]. Despite the corrected attribution of this cell line over 15 years ago, the damage has nonetheless been done; 30% of the above-mentioned references have utilized HepG2 cells in the context of HCC, including nearly 1200 references since 2023 alone.

### 2.4. Huh6 Cells

Huh6 cells represent the next most commonly used human HB cell line and the only other one that is commercially available. Like HepG2 cells, at least two clonal derivatives of this cell line, HLM-3 and Clone 5, have been derived that show increased metastatic behavior in immuno-compromised mice [40,57]. Huh6 cells also possess a mutant form of CTNNB1, albeit a missense (G34V) rather than a deletion mutation [36].

### 2.5. Molecular Heterogeneity of Human HB Cell Lines

The fact that >99% of published studies with human HB cell lines have used either HepG2 or Huh6 cells further underscores the dire need for additional ones as first pointed out by Rikhi et al. [31]. However, even if these were available, several seemingly insurmountable problems would remain. First, even though the mutational landscape of HBs is rather limited [58,59], it is unlikely that any two tumors would be molecularly identical. Previous analyses of both human and experimental murine HBs have shown that the nature of the underlying oncogenic drivers can profoundly impact survival and the behaviors of both the tumors and the cell lines derived from them [10,11,12,33,60,61]. Indeed, even different CTNNB1 mutations exert non-identical effects on tumor growth and behavior in murine models of HB [60,62]. This likely reflects the unique stability, nuclear accessibility and transcriptional range and potency of each mutant protein. Second, somatic genetic heterogeneity and ethnicity among different individuals with HB could influence tumor behaviors, although the degree to which this occurs is likely small [63]. Lastly, the utility of all human cell lines or patient-derived HB xenografts for modeling tumor growth in vivo is severely limited by the fact that they require propagation in immuno-deficient mice [64,65,66]. This naturally impacts the ability to study recently appreciated roles of the immune system in HB tumorigenesis and how it can be harnessed for various immunotherapies [67,68,69,70,71].

### 2.6. Cell Lines Derived from Patient-Derived Xenografts (PDXs)

A number of recent publications have described the establishment of PDX models of human HBs [27,72,73,74,75,76,77,78,79]. However, with one or possibly two exceptions, none of these appear to involve the derivation of immortalized cell lines. Those described by Kats et al. [78] originate from Xentech, a French biotech company that has developed numerous human HB PDX models and, from these, at least 35 cell lines. Based on information for nine of these lines (https://www.xentech.eu/models/pdx-derived/ (accessed on 1 November 2025)), they appear to be of a proprietary nature and available to outside investigators only for in-house drug screening projects. Furthermore, the means by which these cell lines were immortalized is not provided. Eight of the cell lines are described as possessing unspecified mutations in the *CTNNB1* gene and no further molecular characterizations are provided. “HB-17” cells represent another possible human HB cell line derived from a human PDX model by Espinoza et al. [74]. However, it is not clear if these are truly immortalized and to our knowledge, they have not been reported in any other publications. The single human HB cell line mentioned by Demir et al. [72] was obtained from Xentech, two of whose employees were co-authors on the paper. Finally, and as mentioned throughout our paper, all human HB cell lines, regardless of whether they were derived from primary tumors initially or from PDXs subsequently, still represent unique molecular situations, cannot provide bespoke genetic backgrounds and are poorly suited for studies that aim to examine interactions with the host immune system.

### 2.7. The Oncogenic Drivers of Human HB

Since HB is the least genetically complex of all human cancers [58,59], primary murine tumors and cell lines expressing the most common oncogenic drivers will be representative of a larger fraction of human tumors and will thus be of proportionately greater utility. A relatively small number of cell lines should thus suffice to create a reasonably complete library of the most common HB molecular subtypes.

Consistent with HB’s genetic simplicity, only a handful of recurrent mutations or dysregulated oncogene and tumor TS pathways have been identified in human HBs at frequencies exceeding ~5% (Table 2) [51,80,81,82]. The most common of these involves heterogeneous missense or in-frame deletion mutations in exon 3 of the *CTNNB1* gene, which encodes the CTNNB1 (β-catenin) transcription factor (TF). HB-associated *CTNNB1* gene mutation rates as high as 90% have been identified in some studies although the incidence in Asian populations is reported to be only half that seen in Caucasians [13,38,51,53,54,60,63,80,81,83,84,85]. The common feature of these mutations is that they abrogate the normal, Wnt ligand-dependent interaction between CTNNB1 and the “APC” TS complex, which sequesters CTNNB1 in a transcriptionally inactive cytoplasmic state. Mutant CTNNB1, unable to interact with the APC complex, instead become Wnt-independent, constitutively translocate to the nucleus, and activate a large number of genes involved in cell cycle control, survival and migration, notably *MYC* and *CCND1* [51,60,86,87,88,89].

The HIPPO/YAP/TAZ pathway is perhaps the next most commonly de-regulated one in HB (Table 2) [60,62,88,90,91,92,93]. Although recurrent mutations have not been reported in this pathway, the wild-type YAP (yes-associated protein) TF is over-expressed and constitutively localized to the nucleus in 50–60% of primary HBs [88,90]. Moreover, HBs in mice can be induced following the enforced over-expression in the liver of a patient-derived mutant form of CTNNB1 (del90) and an engineered, phosphorylation-defective form of YAP (YAP^S127A^) that constitutively localizes to the nucleus and recapitulates the nuclear localization of wild-type YAP in human HBs [88,90,94]. These studies also established that HB induction requires the cooperation between mutant forms of CTNNB1 and YAP^S127A^ and that neither one alone is oncogenic [60,86,88].

**Table 2:** Most frequent primary oncogenic drivers in human hepatoblastoma.

Name of Driver	Function	% of Tumors	Form of Deregulation ^1^	Reference(s)
β-catenin (CTNNB1)	Oncogene	~60–80	PM, DEL	[10,38,53,54,60,80]
Hippo/YAP/TAZ	Oncogene	~50–60	O	[51,88,90,93]
NRF2/NFE2L2	Oncogene	~50	CNV, O, PM	[13,80,89]
TERT	Oncogene	6	pMut ^2^	[80,81]
MYC	Oncogene	~25–50	CNV, A, OE	[10,14,43]
APC	TS	15	T	[91,95,96,97]
AXIN1/2	TS	8	PM	[56,98,99]
ARID1A	TS	6	O	[81]
CDKN2A	TS	50	pMe, O	[32,100,101]

^1^ PM: point mutation; DEL: deletion mutation; O: other; CNV: copy number variation; pMut; promoter mutation; A: amplification; OE: overexpression; T: truncation; pME; promoter hypermethylation, ^2^ Largely confined to tumors from older individuals (>8 years).

Deregulation of the *NRF2/NFE2L2 (NRF2)* gene or protein is observed in about half of human HBs (Table 2). About 90% of the former involve gene copy number increases as high as 6-fold whereas the remaining ones involve recurrent point mutations, usually in residues residing within or in close proximity to the so-called DLG and ETGE motifs centered around residues 23–31 and 77–82, respectively [13,80,89,102]. Other cases of HB do not involve qualitative or quantitative changes in the NRF2 gene. Rather, as mentioned above for YAP, some tumors show constitutive nuclear localization of NRF2 [4]. Like CTNNB1 and YAP, NRF2 is a TF that is normally maintained in an inactive state in the cytoplasm via a redox-sensitive association with its inhibitor, KEAP1 [61,103]. Oxidative or other xenobiotic stresses promote NRF2-KEAP1 dissociation leading to NRF2’s translocation to the nucleus and the induction of numerous genes involved in the anti-oxidant response [51,103,104,105,106]. Previous studies had suggested that NRF2 contributes to cancer only indirectly and that this could either accelerate or delay its onset depending upon when during the transformation process its deregulation occurred [51,102,106,107]. For example, mutations acquired early would lead to a chronically activated anti-oxidant response, less oxidative DNA damage, long-term reduction in the acquisition of potentially oncogenic mutations, and a slowing of tumor evolution. In contrast, *NRF2* mutations acquired at later times would allow already activated oncogenes that are associated with reactive oxygen species (ROS) production such as *MYC* and *RAS* to be expressed at even higher levels and drive more aggressive tumors without succumbing to ROS-mediated toxicity [108,109,110]. However, we have demonstrated that the over-expression of patient-derived mutant forms of NRF2 (specifically NRF2^L30P^ [N] and NRF2^R34P^) can cooperate with CTNNB1^del90^ (B) or YAP^S127A^ (Y) to induce HBs with near 100% efficiency [89]. Each pair-wise combination of B, Y and N (BY, BN and YN) generates HBs with distinct biological behaviors and transcriptional profiles and the triple “BYN” combination generating particularly aggressive tumors.

Mutations in the promoter of *TERT* (telomerase reverse transcriptase), specifically at two mutually exclusive hotspots (positions −124 and −146 upstream of the ATG start site), have been described in about 6% of HBs in contrast to HCCs where such mutations are about ten times more frequent [80,81,111,112]. These C→T transitions generate de novo binding sites for ETS family TFs, leading to *TERT* reactivation in cells in which it had been previously quiescent. Current models indicate that, while not directly transforming, *TERT* reactivation allows cells to overcome the Hayflick limit and achieve immortalization [25,113,114].

The *MYC* gene is commonly over-expressed in HB [14,86]. Much of this is likely indirect and a reflection of aberrant signaling directed by the above-mentioned oncogenes and the high proliferative rates of the tumor cells [115,116,117,118,119]. However, additional modest up-regulation may result from gains in chromosome 8, which harbors the *MYC* gene and which occurs in 25–50% of HBs [10,14,43]. On the other hand, a more recent assessment of 112 HBs has reported no such increase in *MYC* copy number [81].

Mutations in three distinct TS pathways are associated with >5% of HBs (Table 2). The first involves various subunits of the APC TS complex, which are exclusively associated with HBs that harbor wild-type *CTNNB1* loci. Initially thought to comprise a relatively small fraction of HBs, as many as 15% of sporadic tumors may in fact harbor mutations in APC itself or other members of the APC complex, notably Axin1 and Axin 2 (Table 2) although not all of the latter’s missense mutations have been shown to be associated with a loss of TS function [56,82,91,95,96,97,99]. Nonetheless, this would be consistent with HB mouse models showing that sufficiently high levels of wild-type CTNNB1 (in association with YAP^S127A^) are in fact oncogenic. In this capacity, they can overwhelm the cytoplasmic retention capacity of the APC complex, enter the nucleus and deregulate transcription in much the same way as mutant forms do [60]. Collectively, these results suggest that mutations in CTNNB1 or a component of the APC complex are associated with well over 80–90% of human HBs and that CTNNB1 is a major driver of the disease. *APC* mutations are invariably associated with HBs that develop in individuals with familial adenomatous polyposis (FAP); while classically associated with the development of thousands of colonic polyps that have a high rate of malignant transformation, individuals with FAP also have as much as a 7500-fold higher than average risk of developing HB as children [120]. Because of the life-long implications of FAP for both patients and their immediate relatives, it is currently recommended that all children with HB be screened for *APC* mutations, although it can be argued that this be reserved for those individuals whose tumors harbor wild-type *CTNNB1* genes [32,95].

The second TS pathway involves the *CDKN2A* gene in which promoter hypermethylation, variously sized deletions or other uncharacterized forms of transcriptional silencing have been described in approximately half of human HBs (Table 2) [32,100,101,121]. This intensely studied locus encodes two well-known TSs, p16^INK4A^ and p14^ARF^ (p19^ARF^ in the mouse). Their transcripts are initiated from alternate promoters associated with different first exons, 1α and 1β, with the former residing ~20 kb downstream of the latter. Splice acceptors for these alternate exon 1 transcripts, located at different sites in exon 2, generate downstream mRNAs that are identical in sequence but differentially translated in alternate reading frames such that the two proteins show no relatedness [122]. 16^INK4A^ is an indirect TS in that its up-regulation leads to retinoblastoma protein (Rb)-mediated growth suppression and the induction of senescence [122,123]. p19^ARF^ also exerts its TS effects indirectly via its up-regulation of TP53-mediated growth arrest and apoptotic signaling. Considerable tissue-specific cross-talk and functional overlap exist between these two pathways. Which of these is more important for achieving immortalization when inactivated is thus highly context-dependent although they probably act additively if not synergistically given that their expression is highly correlated and that deletion of the entire *CDKN2A* locus, rather than just exon 1α or 1β is a common occurrence in a number of human cancers [124,125,126,127,128].

Finally, nonsense and frameshift mutations in the *ARID1A* TS gene have been described in about 6% of HBs, which is about one-third the frequency seen in HCCs [81,129,130]. As a component of the SWI/SNF chromatin remodeling complex, ARID1A plays a key role in directing SWI/SNF to enhancers and ensuring that the correct epigenetic landscape and optimal levels of target gene expression are achieved [130,131,132]. Recurrent mutations in other members of the SWI/SNF complex resulting in both gain- and loss-of-function have been detected in many cancers although those involving ARID1A are the most common [133].

## 3. Murine HB Cell Lines: Recent Progress and the Meeting of (Some) Unfulfilled Needs

### 3.1. Background

As noted above, in vitro studies of HB have, until quite recently, been limited by a paucity of established cell lines and an over-reliance on HepG2 cells. As is true for human cell lines from any cancer, “what you see is what you get” in that researchers must be content with cell lines bearing driver mutations that cannot be pre-selected and may not necessarily be representative of a molecular group or transcriptional landscape of special interest. The use of such cell lines for pre-clinical testing might therefore either under- or over-estimate the actual value and importance of new drugs or drug targets for tumors with different driver mutations [19]. Finally, because their in vivo propagation requires an immuno-compromised host, no human cell line can overcome their inherent inferiority for studies involving tumor-immune system interactions [67,69,70,134]. Consequently, and despite not being of human origin, murine-derived HB cell lines offer a number of distinct advantages. We discuss below the various types of murine HB cell lines that are currently available to investigators and/or that can be readily generated, along with the advantages and disadvantages of each (Table 3).

### 3.2. Chemically Induced Murine HB Cell Lines

The MHB-2 “HB” cell line, the first to be described, was generated from a murine liver cancer that arose following treatment with diethylnitrosamine (DEN) and phenobarbital (PB) in which the overall incidence of tumor formation was stated to be 29% but the efficiency of cell line derivation was not reported [61,135,136]. Described as a HB tumor that also expressed biliary markers, this derivative line did not form subcutaneous tumors in the mouse strain of origin. Subsequent studies with tumors induced with only DEN in other mouse strains have shown them to more closely resemble HCCs, which would be consistent with the finding that they do not generally appear before ~20 months of age, i.e., in older mice (Table 3) [137,138]. Interestingly, all tumors examined contained activating missense mutations in *Hras* (codon 61), *Braf* (codon 584) or *Egfr* (codon 254), which are not as a rule found in human HBs or even in HCCs, although EGFR is often over-expressed in the latter in the absence of gene amplification [139,140,141]. On the other hand, ~20% of DEN only-induced tumors originating in older animals do contain *Apc* or *Ctnnb11* mutations. It is not clear whether MHB-2’s original description as arising from a HB is due to a mouse strain difference, a mis-diagnosis of the original tumor, the use of PB as a tumor promoter [142] or simply a rare occurrence in which a HB rather than a HCC was induced. Regardless, because the cell line is not tumorigenic and carries mutations with no known relationship to human HBs, it serves as a questionable and limited model. Moreover, neither it nor other DEN-induced tumors are histologically or molecularly representative of naturally occurring human HBs or of murine HBs driven by the enforced over-expression of known oncogenes as described below.

### 3.3. Genetically Defined Murine HB Cell Lines: Enforced Over-Expression of Mutant Forms of CTNNB1, YAP and NRF2

Murine HBs resembling the human crowded fetal subtype can be generated using hydrodynamic tail vein injection (HDTVI) to deliver a mutant form of CTNNB1^del90^ (B) and YAP^S127A^ (Y) encoded by Sleeping Beauty (SB) plasmid vectors [86,88]. Although *MYC* is not necessary for the proliferation of normal hepatocytes or the initiation of these tumors, it is needed to sustain maximal tumor growth rates [86,143,144,145,146]. The finding that liver cancers can be initiated by over-expressing *MYC* alone suggests that its cryptic oncogenic tendencies emerge only when it is aberrantly over-expressed and engages low-affinity target genes that are normally not subject to its control [20,147,148,149,150,151].

We subsequently reported that patient-derived NRF2 mutants NRF^L30P^ (N) and NRF^R34P^ were also oncogenic when co-expressed with B or Y. Pairwise combinations of these three drivers generated distinct tumor types that we designated as BY, BN and YN [89]. All three pairwise combinations generated tumors with near 100% efficiency although BY tumors grew significantly more rapidly. A fourth tumor group, BYN, generated by all three mutant oncoproteins, drove the most rapidly growing tumors with the same crowded fetal histology as BY tumors but also with innumerable fluid-filled cysts many of which abutted small, well-demarcated regions of necrosis [89]. These mimicked a rare cystic form of human HB [152,153]. In >30 attempts, we failed to generate immortalized cell lines from any of these different HB groups.

p16^INK4a^ and/or p14^ARF^/19^ARF^, or their downstream TSs, Rb and TP53, are frequently down-regulated, inactivated or mutated in human and murine tumors, including HBs [100,101,123,125,127,154,155]. Similarly, most human HBs that we examined showed undetectable or extremely low-level p16^INK4A^ and p14^ARF^ transcript expression [32]. In contrast, some human HBs and all four B/Y/N murine HB subtypes expressed p16^INK4a^ and p14^ARF^/19^ARF^ at high levels. Many relevant downstream transcriptional targets of Rb and TP53 were also dysregulated in ways that indicated they were competing with and overriding the TS’s pro-senescent, pro-apoptotic and growth-inhibitory signals. Yet, these pathways remained intact as evidenced by the ability of over-expressed wild-type p16^INK4A^- or p19^ARF^ to block de novo tumorigenesis [32,156]. Seemingly paradoxical up-regulation of p16^INK4A^ and/or p14^ARF^ occurs in many different human tumor types and is often associated with shorter survival [155,157,158]. Reasoning that silencing or mutating *Cdkn2a* might facilitate in vitro immortalization as it does for other normal and transformed cells, we used in vivo Crispr/Cas9-mediated targeting of *Cdkn2a*’s exon 2 at the time of tumor initiation [25,122,123,126,159]. This allowed us to generate immortalized HB cell lines in 16 of 16 attempts (Table 3) [32,33].

BY and BYN immortalized cell lines (eight BY and three BYN) were the first to be characterized [32]. Unlike their non-targeted counterparts, Crispr-targeted BY and BYN HBs expressed little to no wild-type p16^INK4A^ and p19^ARF^ proteins. This remained so after cells from the primary tumors were plated in vitro. These non-proliferating cells, referred to as “P0” cells, contained numerous large, non-coding *Cdkn2a* deletions and an average of 37 other mutations encoding various missense, truncated or fused p16^INK4A^ and/or p19^ARF^ proteins. The pooled proliferating clones that eventually emerged from the P0 population several weeks later demonstrated negative selection for ~40% of these initial mutations with as few as one or two comprising >50% of the entire population in seven of the nine cell lines examined. As with primary tumors, the proliferation of these cells could also be markedly inhibited by re-expressing wild-type p16^INK4A^ or p19^ARF^ [32]. In nearly every case, enforcing the expression of some of the more dominant of the above-mentioned selected mutants also suppressed growth as efficiently as wild-type p16^INK4A^ and p19^ARF^, if not more so, thus again confirming the intactness of the downstream Rb and TP53 pathways.

BY and BYN cell lines largely retained the features of the original tumors from which they originated. For example, all except BY2 cells generated rapidly growing subcutaneous tumors resembling primary crowded fetal HBs and did so without the need for exogenous basement membrane preparations such as Matrigel. Although primary tumors were not metastatic, widespread pulmonary “pseudo-metastases” could be generated within 3–5 weeks after standard tail vein delivery of tumor cells.

Using the approach described above, two BN and three YN cell lines were next established [32,33]. One BN and all three YN lines could also be propagated as subcutaneous tumors and pulmonary pseudo-metastases following tail vein injection. All retained the histologic appearance of the original primary tumors, with BN tumors resembling differentiated HBs and YN tumors resembling HBs with variable HCC-like features. They also expressed an assortment of p16^INK4A^ and p19^ARF^ mutants different from those seen in BY and BYN tumors [32,33]. Moreover, enforcing the expression of BY- and BYN-associated p16^INK4A^ and/or p19^ARF^ mutants in BN and YN cells often had quite different consequences, with some of the mutants being just as growth suppressive and others being either more or less so. Collectively, these findings strongly suggested that the driver oncoprotein backgrounds significantly influence the nature of the *Cdkn2a* mutant(s) that will ultimately achieve clonal dominance and the degree of growth suppression that each will exert.

An unexpected feature of BY and BN cell lines, but particularly the latter, was discovered while investigating their ability to form and survive as tumor cell spheroids on non-adherent surfaces [160]. Despite developing hypoxic interiors, these spheroids remained highly viable for at least 2–3 weeks and rapidly resumed monolayer growth after re-plating into standard tissue culture dishes. Because hypoxic tumor cells can sometimes acquire endothelial cell (EC)-like properties [161,162,163], this was investigated for the HB cell lines by stably incorporating into them a vector encoding EGFP under the control of the EC-specific TIE2 promoter [161,162]. Significant EGFP expression was detected in the hypoxic interiors of spheroids within 3–5 days of their formation whereas in monolayer cultures, up to 5% of the BN cell population showed EGFP expression within 24–48 h of exposure to 1% oxygen. In the latter case, the ~25 day half-life of EGFP expression upon returning to a normoxic environment provided sufficient time for EGFP+ cell populations to be isolated by fluorescence-activated cell sorting, expanded, and examined for various EC attributes. These cells rapidly and efficiently formed EC-like “tubes” following their return to hypoxic conditions and did so without the basement membrane support and EC-specific growth medium that is normally required [164]. EGFP+ BN cells also formed tumors much more rapidly than normoxia-maintained control cells. By the time these cells formed tumors, they no longer expressed EGFP but did contain 1.7-fold more tumor-associated small blood vessels while maintaining their HB-like histology. This suggested that the more rapid growth of these tumors was due to a more robust vasculogenic activity during the earliest stages of tumorigenesis [165].

To determine the relevance of the above findings and to provide in vivo context, the transcriptomes of normal murine livers and each of the four molecular groups of primary HBs were examined for the expression of 1853 non-redundant EC-specific transcripts derived the Molecular Signatures Database [33]. Over half the constituent gene sets were obtained from ECs of fetal or embryonic tissues and from multiple diverse tissues [166,167,168]. Each HB group expressed distinct subsets of these EC-specific genes, with those from BN tumors most closely matching those of normal livers and the other three profiles more closely resembling one another and showing marked dissimilarity to the profiles of livers. In these cases, a set of ~600 transcripts that is highly enriched in liver-associated ECs was markedly down-regulated and replaced by EC-specific transcripts from the non-liver sources. Thus, with the exception of BN HBs, the EC-specific gene expression profiles of the other B/Y/N HB groups derived from a variety of non-liver-associated EC-specific genes. Human HBs also displayed EC-specific gene expression profiles that were distinct from those expressed by livers. The greater heterogeneity of these gene expression profiles relative to murine HBs likely reflected their greater molecular heterogeneity [11,12].

Differential gene expression was examined in BN cells maintained under four strictly controlled conditions in vitro: 1. Continuous normoxia (UN); 2. Exposure to hypoxia for two days (UH); 3. The same conditions as in (2) except that EGFP+ cells were sorted and expanded under normoxic conditions for ~ten days (SN); 4. The same conditions as in (3) except that the sorted cells were returned to a hypoxic environment for two days (SH). Among the more notable findings were that the SN and SH groups continued to express ~300 hepatocyte-specific genes in addition to the EC-specific ones. This and the reversible nature of the EC-like phenotype strongly suggested that these cells originated from a subpopulation of transformed hepatocytes rather than from a more specialized EC-like precursor population. Also of note was that the above four cell groups each expressed distinct subsets of the previously mentioned EC-specific genes. Together with the results from primary HBs, these findings indicated that the striking acquisition of EC-like properties by BN cells correlates with the ectopic induction of EC-specific genes that are not normally expressed by the liver. The extent and success of the epithelial (hepatocyte)-to-mesenchymal (EC) trans-differentiation process thus seemingly reflects the degree to which these genes can cooperate to generate a chimeric but functional EC population that contributes to the formation of a neo-vasculature.

Subsequent interrogation of the above EC-specific genes was performed in mice whose hepatocytes expressed a doxycycline-responsive human *MYC* transgene (Tet-*MYC* mice) and rapidly develop HCCs with HB-like characteristics [147,149]. As previously noted, about one-third of the above 1853 EC-specific genes were expressed in control livers and at early times after *MYC* induction [32,147,149]. The large neoplasms arising ~30 days later were associated with down-regulation of these genes and up-regulation of a new subset of the transcripts. These also disappeared within three days of the onset of tumor regression and were replaced by a third set of EC-specific transcripts that persisted until at least day seven. Recurrent tumors were associated with the suppression of this regression-specific transcript subset and the re-expression of those detected in the original primary tumors. Together with the results from BN cell line studies, these findings indicate that the complement of EC-specific genes associated with primary and recurrent liver tumors is both very different from that seen in control livers and distinct from that seen during the early stages of tumor regression when substantial vascular remodeling and retreat is likely occurring. Different EC-specific genes thus appear to be recruited in response to the specific needs of mature and regressing tumors. EC-specific genes were also differentially expressed in eight other human cancer types and their corresponding normal tissues. In seven of these cases, tumors expressing the highest levels of these transcripts were associated with significantly shorter patient survival times [33].

To explain why BN cells, more so than the others, trans-differentiated more efficiently into ECs [33]. It was speculated that Y could suppress the EC program as reported in other cell types [169,170]. Indeed, stably expressing Y in BN cells (and converting them to BYN cells) suppressed their ability to undergo EC trans-differentiation in response to hypoxia by 20-fold [33].

The final question that was addressed was whether cell lines from the above four molecularly defined HB groups could be used to demonstrate differential sensitivities to four drugs commonly used to treat HB (vincristine, etoposide, cis-platinum and doxorubicin). For each agent, all cell lines generally showed similar sensitivities thereby suggesting that factors other than B, Y and N combinations determine differential responses to these drugs in vivo.

### 3.4. Genetically Defined Murine HB Cell Lines: Enforced Over-Expression of MYC

Liver cancers can be efficiently induced in the above-described Tet-*MYC* mice or in those that constitutively express a human *MYC* transgene under the control of a synthetic “CAG promoter” that initiates transcription in mid-gestation (ABC-*MYC* mice) [20,147,149]. Although tumors in both models show high expression of MYC protein, their levels have not to our knowledge been directly compared. While possibly due to differences in expression levels or mouse strains, the somewhat shorter median survival associated with ABC-*MYC* tumors more likely reflects the fact that their MYC up-regulation is initiated prior to birth (~E7.5–9) whereas Tet-*MYC* induction has generally been delayed for as long as 4–6 weeks after birth. Fetal livers also contain a greater proportion of the hepatic progenitor target cells from which HBs are believed to arise [5,171,172]. These *MYC*-driven tumors have been variously described as moderately differentiated HCCs with HB-like characteristics (Tet-*MYC*) or fetal/embryonal HBs (ABC-*MYC*) [20,147,149]. Similar HCC-like tumors have been described as arising at variable frequencies following HDTVI-mediated delivery of MYC alone or in combinations with activated forms of Akt or YAP [173].

The liver cancers that develop in response to *MYC* over-expression appear quite rapidly [20,147,149]. This contrasts with the conventional wisdom that a single driver, whether it be an oncogene or a TS, is not tumorigenic unless accompanied by at least one additional “hit” that is provided either experimentally or by chance occurrence, with the latter generally being associated with a longer lag phase [88,174,175,176,177,178,179,180]. Although neither *CTNNB1* nor *YAP1* is known to be a direct transcriptional target for MYC, both genes are over-expressed in the above-described MYC-driven tumors [20,147]. Functionally, therefore, these tumors may be more related to B/Y/N tumors than initially meets the eye. It may also explain why, at the whole transcriptome level, these tumors resemble HBs [20]. The lack of any known *CTNNB1* mutations in these tumors does not rule out an oncogenic role for the wild-type protein any more than it does in HBs with mutations involving components of APC complex or in mice that over-express the wild-type protein [42,56,60,91,95,96,97,98,99]. So long as CTNNB1 is expressed at sufficiently high levels to overwhelm its inhibitory cytoplasmic interaction with APC, it can freely enter the nucleus and subsume a role in oncogenesis [60].

Our own previously mentioned failure to establish immortalized cell lines from any of the four genetic groups of B/Y/N HBs without concurrently targeting the *Cdkn2a* locus [32,33], raises the question of how to account for the ease with which multiple cell lines could be generated from ABC-*MYC*-driven HBs [20]. This is a particularly relevant question given that, as suggested above, B/Y/N-induced tumors, which express high levels of Myc appear to be functionally related to ABC-*MYC*-induced tumors, which express high levels of B, Y and N [20,32,60]. The explanation likely lies with the *MYC* transgene, which is both aberrantly deregulated and expressed at much higher levels in these tumors than is endogenous *MYC* in B/Y/N tumors. MYC is a well-known immortalizing factor although the precise mechanisms by which this is achieved are likely multifactorial, tissue-specific, and finely balanced with its promotion of apoptosis and senescence via regulation of p16^INK4A^, p19^ARF^ and other genes involved in aging and survival [181,182,183,184,185,186,187,188,189].

Fang et al. have more fully characterized ABC-*MYC* tumors and several of the immortalized cell lines originating from them [20]. As was true for all four B/Y/N and Tet-*MYC* tumor groups, the multi-focal primary ABC-*MYC* neoplasms resembled human embryonal or mixed embryonal-fetal HBs although focal subpopulations of cells resembling fetal and cholangioblastic HBs and HCC were noted as well and were most consistent with the mixed epithelial subtypes of human HB. Although no individual immuno-histochemical markers can clearly distinguish between HB and HCC, ABC-*MYC* tumors showed combined staining profiles that were more consistent with HB, i.e., AFP+, glypican-3+, glutamine synthetase+, Sal4+, and arginase-1+. On the other hand, although ABC-*MYC* tumors showed strong staining for CTNNB1, it was largely confined to the cytoplasm, thus indicating that the mechanisms of transformation were distinct from those of B/Y/N-type murine HBs, where CTNNB1 is nuclear-localized in all cases as it is in the majority of human HBs as well [38,53,54,56,60,88,90]. Because neither the level nor localization of YAP and NRF2 were described, the degree to which these contributed to tumorigenesis could not be ascertained. Fang et al. also speculated that the histologic diversity of ABC-*MYC* tumors could be explained by the degree to which *MYC* was overexpressed and/or the stage of differentiation of different hepatocyte progenitor target populations [20]. Similar arguments could explain the heterogeneity observed among Tet-*MYC* and different B/Y/N tumor types.

A better definition of the HB-like properties of ABC-*MYC* tumors was obtained by unbiased transcriptome profiling and gene set enrichment analysis (GSEA), which revealed a close relatedness to human HBs [20]. This analysis also showed evidence for up-regulation of the CTNNB1 signaling pathway despite the fact that, as mentioned above, the tumors did not show immuno-histochemical evidence of nuclear CTNNB1 accumulation. Overall, 50% of up-regulated genes and 43% of down-regulated genes in ABC-*MYC* tumors overlapped with their counterparts in primary human HBs, indicating a high degree of similarity among neoplasms from these different species. Functionally, the deregulated genes in murine HBs could be grouped into pathways that are known to be associated with MYC-regulated genes, including those pertaining to cell cycle, DNA replication and repair and RNA splicing [190,191]. Further GSEA profiling showed that ABC-*MYC* tumors also up-regulated genes associated with cancer stem cells and “undifferentiated cancers” while confirming their membership in the HB molecular category known as “C2” that is associated with unfavorable outcomes [10,11,12,13,20,89,192].

At least six cell lines from ABC-*MYC* tumors were generated in much the same way as described above for B/Y/N HB tumor groups except that single-cell suspensions were first allowed to form tumor cell spheroids on non-adherent surfaces before being transferred to standard tissue culture plates where they were subsequently maintained as monolayer cultures. These cells could again form spheroids when transferred back onto non-adherent surfaces and retained their tumorigenic capacity when propagated subcutaneously or as pulmonary pseudo-metastatic growths (Jun Yang, personal communication) [20].

Genome-wide Crispr-Cas9 screening performed in one of the above ABC-*MYC* cell lines identified nearly 1600 genes deemed essential for survival and/or proliferation, with 100 of these encoding targets with known inhibitors. Among the most functionally relevant genes were those involved in Hippo/YAP signaling indicating that, as in the case of B/Y/N tumors and cell lines, this pathway, like the WNT/CTNNB1 pathway, is deregulated by *MYC* over-expression. Genes deemed as being essential in this screen included *Yap*, *Taz*, *Taok1*, *Lats1*, and *Nf2.* Importantly, the *Cdkn2a* locus, which is so important for the establishment of B/Y/N cell lines [32,33], was also among the negatively selected genes. Crispr-Cas9 screens performed in two additional independently derived ABC-*MYC* cell lines identified 1346 and 846 essential genes, with 471 being shared among the three cell lines. This indicated that, even with this simple model of HB generation, significant genetic heterogeneity and differential dependencies still existed among cell lines derived from independently generated tumors driven solely by *MYC*.

Comparing the three ABC-*MYC* cell lines from the above studies to those previously identified in a similar Crispr-Cas9 screen performed in human Huh6 HB cells [19] revealed a remarkable 72% overlap among the cancer dependency genes, most notably those again involving YAP signaling [20]. This confirmed that oncogenic and therapeutically targetable genes were shared between human and murine HBs. It also provided validation that these cell lines could be of considerable value in the search for novel molecular susceptibilities.

A genome-wide Crispr-Cas9 screen was next performed in one of the cell lines in combination with IC_20_ and IC_90_ doses of doxorubicin, a drug that is commonly used to treat HB [193]. Depending on the doxorubicin dose, between 70 and 335 genes were identified in which mutation/inactivation were associated with increased drug sensitivity or resistance. In the latter case, these included *Cdkn2a* (as well as *Tp53*), in keeping with the known effects of p16^INK4A^ and p19^ARF^ on senescence and apoptosis noted above for B/Y/N tumors. This also suggested that, unlike the case in which *Cdkn2a* needed to be deliberately targeted during the generation of B/Y/N cells, the locus remained intact during ABC-*MYC* cell line derivation and that the basis of their in vitro immortalization was distinct. Other genes noted as modifying doxorubicin sensitivity included those involved in DNA repair; non-homologous end-joining; and mToR, PI3 kinase and MEK signaling. Cdk7 and Aurora A kinase were also identified as potential targets.

The afore-mentioned ABC-*MYC* cell line was next used to test 125 FDA-approved cancer drugs, 51 of which inhibited viability by >50% [20]. Consistent with the findings from the previous high throughput Crispr-Cas9 screen, active drugs (in addition to doxorubicin) included known inhibitors of mTOR, MEK, EGFR, CDK7 and Aurora kinase. In several cases, these findings were consistent with previously reported small clinical trials suggesting that such inhibitors might be useful in the treatment of either primary or relapsed/refractory HB [85,194,195].

Notable synergy was seen between doxorubicin and the PRKDC inhibitor AZD7648 as well as an anti-PRKDC siRNA. PRKDC (DNA-dependent protein kinase catalytic subunit) plays a critical role in the repair of double-stranded DNA breaks (DSBs) and thus might be expected to participate in the repair of the DSBs induced by a topoisomerase inhibitor such as doxorubicin [196]. Importantly, similar synergy was observed in two other ABC-*MYC* cell lines as well as with a second, structurally distinct PRKCD inhibitor, NU7441.

## 4. Relatedness of Murine HB Cell Lines

### 4.1. Genetic Relatedness

To ascertain the degree to which each of the above-discussed primary groups of tumors differed from one another, we compared their whole transcriptomic profiles. A high degree of similarity was observed across all six groups, thus reaffirming their previously discussed HB-like properties (Supplementary File S2, Figure S1) [20,32,147]. A minor exception was seen with a group of ~400 transcripts that was more markedly down-regulated in ABC-*MYC* tumors versus all other groups (*p* < 10^−15^ in all cases using a two sample *t*-test) and that was weighted in favor of those encoding proteins involved in steroid and glycogen metabolism. Also seen was a highly significant (ca. two-fold) increase in Prkdc transcripts in ABC-*MYC* tumors that may have accounted for the enhanced sensitivity of their derivative cell lines to inhibitors of this enzyme as noted in the previous section [20]. A more modest (ca. 1.5-fold) increase in Prkdc transcripts was seen in BYN HBs but not in BY, BN or YN tumors (Supplementary File S2, Figure S1B). Importantly, elevated Prkdc transcripts were not observed in Tet-*MYC* tumors. The reasons for these inter-group variations are unclear but could be due to non-mutually exclusive differences in mouse strains and/or target cell populations, to the levels and interplay among the B, Y and N drivers, and to differences in the levels of MYC over-expression [32]. These studies, while exciting, should still serve as a cautionary note. Though indicating that murine HB cell lines will likely be highly useful for identifying novel targets and for the screening of new drugs [20], they also strongly imply that the importance of such findings may well depend not only upon the underlying molecular subtype of the cell line(s) as we originally proposed but also on inter-cell line variations over which there is little or no control [33]. Multiple HB cell lines representing a variety of different molecular subtypes are thus likely to be needed in order to gain a comprehensive understanding of which human HBs show the best responses to new chemotherapeutic agents and allow for the creation of more personalized therapies. It is also possible that additional independently derived cell lines of each individual group will be needed to account for unavoidable intra-group differences.

### 4.2. Post-Translational Relatedness

In human and/or experimental murine HBs driven by different B/Y/N combinations, both wild-type and mutant forms of CTNNB1, YAP and NRF2 localize to nuclei although the extent to which this occurs for CTNNB1 reflects the identity of the mutation [55,60,88,89,197]. In ABC-*MYC* tumors in contrast, endogenous CTNNB1, though over-expressed, was largely confined to the cytoplasm [20]. This raised the question as to how, if at all, β-catenin cooperates with MYC to promote transformation [20]. To extend and expand this observation to the closely related tumors generated in Tet-*MYC* mice (Supplementary File S2, Figure S1A), we separated nuclear and cytoplasmic fractions from tumors and control livers and performed immunoblotting for CTNNB1, YAP and NRF2 as previously described [60]. All three proteins were highly over-expressed in tumors with YAP and NRF2 being confined exclusively to the cytoplasm (Supplementary File S2, Figure S2). In contrast, whereas most CTNNB1 were associated with the cytoplasm, substantial amounts localized to the nuclear compartment. This was consistent with our previous observations in BY HBs generated with various CTNNB1 mutants showing variable amounts of nuclear CTNNB1 [60]. The above findings suggest that, at least in the case of Tet-*MYC* tumors, MYC’s role in transformation may require the cooperation of nuclearly localized and deregulated CTNNB1 but not YAP or NRF2, which remain largely localized to the cytoplasm despite their over-expression. Together with the transcriptional differences noted in Supplementary File S2, Figure S1, the findings further underscore differences between Tet-*MYC* and ABC-*MYC* tumor groups that are likely related to differences in the levels of MYC and/or the underlying identity of the target cell of origin.

## 5. Caveats to the Use of Murine Cell Lines for Drug Screening

While in vitro immortalization of B/Y/N HBs clearly involves the deliberate mutational disruption of the *Cdkn2a* locus, it is less clear how immortalization is achieved in ABC-*MYC*-derived cell lines, although it is likely more complex [20,89]. As previously mentioned, *MYC* deregulation is a well-known means by which primary human and murine cells can be immortalized as is the over-expression of *TERT* and the disabling of *CDKN2A* locus [113,159,181,182,183]. These roads to immortalization connect in circuitous ways by virtue of such features as MYC’s activation of *TERT* and p19^ARF^ and the ability of RB and TP53 to suppress *MYC* [182,183,187,191,198]. Activating these immortalization pathways can profoundly affect chemotherapeutic responses in ways that are tumor-, drug- and context-dependent [199,200,201,202,203,204]. Whether or not any of these pathways are altered in primary tumors will thus likely contribute substantially to chemotherapeutic responses that may or may not reflect those of cell lines derived from them.

Because they are derived from HBs bearing defined oncogenic drivers, ABC-*MYC,* Tet-*MYC* and B/Y/N cell lines very likely lack many of the more minor and/or subtle genetic and epigenetic features associated with naturally occurring human HBs that may affect chemotherapeutic drug sensitivities [58,59,81,82]. The levels of expression of driver oncoproteins, particularly MYC, as well as differences attributable to their being driven by foreign promoters and thus subject to unnatural forms of control, may also lead to the deregulation of downstream target genes that are otherwise normally unresponsive. How these will affect chemotherapeutic drug sensitivities will need to be determined. MYC is a particularly well-studied example of how the expression of its downstream target genes can be highly dependent on the degree to which it is over-expressed and the affinities of the genes’ MYC binding sites [148,151,205]. Similarly, numerous differences in the downstream target genes of CTNNB1 have been described in murine HBs, which differ only in the type of patient-derived CTNNB1 mutant protein they express [60]. Too few studies have been performed to date with the genetically defined murine HB cell lines that have only recently been described [20,24,33]. Future studies will need to be interpreted cautiously and verified in other HB cell lines, organoid and PDX models before proceeding to human trials involving patients whose HBs have been carefully characterized molecularly.

## 6. Advantages and Disadvantages of Human and Murine Cell Lines

### 6.1. Background

As is true for all cell lines, those derived from human and mouse HBs are associated with distinct advantages and disadvantages. Depending upon circumstances, it may be beneficial to favor those derived from one species or to use them in complementary ways such as verifying novel therapeutic susceptibilities or confirming the efficacy of new drugs as previously demonstrated by Fang et al. [20].

### 6.2. Human Cell Lines

Perhaps the greatest advantage of human HB cell lines is simply the fact that they are derived from actual human tumors such that any observed behaviors cannot be mistaken for those that are merely the result of species differences. Human cell lines also contain the full complement of the driver mutations and epigenetic changes that are necessary for tumorigenesis and that have been selected over time both in vivo and in vitro. This contrasts with murine cell lines in which genes such as B, Y, N and MYC are likely to represent only the minimal number of factors necessary to achieve transformation. Finally, human HB cell lines are driven by oncoproteins that, despite their mutated and/or over-expressed state generally remain subject to the control of their endogenous promoters. Oncoproteins such as MYC are notorious for regulating target genes in ways that are highly dependent on driver protein levels as a result of many of these targets possessing low-affinity binding sites [151,206,207]. Tumors arising from the exogenous over-expression of *MYC*, *B*, *Y*, and *N* while technically sharing many of the attributes of actual HBs, might therefore not behave in precisely the same way as their human counterparts, particularly if they regulate a large number of such “pathologic” targets as a result of non-physiologic levels of oncoprotein expression [147,151].

Human HB cell lines also have several disadvantages. First, of course, is their extreme rarity as discussed in Section 3.2, this being a state that has remained unchanged for quite some time and that prompted the search for murine substitutes in the first place [20,31,32,33]. Second, due to genetic drift, sub-cloning and selection for certain traits that favor long-term in vitro growth and survival, human HB cell lines are likely to have acquired properties that are less prominent, if they exist at all, in the tumors from which they originated. Third, human HBs offer no control over the nature and mutation of the driver oncoproteins and TSs. Thus, no two cell lines are likely to be genetically or biologically identical, a situation that is further compounded by the natural genetic diversity within the human population itself [50]. Fourth, because the cell lines (and the tumors from which they originate) already contain the full complement of all the drivers necessary for transformation and other beneficial functions, the individual contributions of these factors can be difficult to establish. Finally, because human cell lines and patient-derived xenografts must be passaged in vivo in immuno-compromised mice, interactions between tumor cells and the immune environment cannot be studied in their natural context [37,66,68,208]. This is a particularly notable weakness given the increasing appreciation for the role played by various immune cell populations in support of HB growth [64,67,69,70,134].

### 6.3. Murine Cell Lines

Compared to human cell lines such as HepG2 and Huh6, murine HB cell lines generated by over-expressing *MYC* or various combinations of B/Y/N offer a number of distinct advantages (Table 4) [20,32,33]. First, and at least in the case of the latter groups, their generation is both easy and efficient. Second, they are easy to maintain and require no specialized growth medium or exogenous additives. Third, they are genetically defined, which provides opportunities to discern the effects of individual or additional oncogenes and/or TSs. An example of this was our previous demonstration that the transition of BY to BYN HBs is marked by more rapid growth and the acquisition of a new histologic profile marked by innumerable fluid-filled cysts with adjacent and well-demarcated areas of necrosis [32]. Fourth, aside from the deliberately introduced drivers, they are isogenic, thereby allowing for the identification and study of random, uncontrollable and unanticipated genetic alterations that impact tumor behavior. Fifth, the ease with which B/Y/N HBs can be generated suggests that other bespoke cell lines bearing different or novel oncogenic drivers could be as well. These could, for example, include lines derived using different patient-derived *CTNNB1* mutations or previously uninvestigated driver genes such as *Tert* or *Arid1a*. In this manner, novel cooperating oncogenes and TSs might be discovered. Sixth, collections of multiple primary HBs and cell lines derived from them provide the ability to identify common downstream targets whose identities can help to pinpoint the precise means by which transformation is achieved. As an example, we performed such experiments in BY, BN, YN and BYN tumors along with BY tumors expressing several different CTNNB1 mutations [60]. 22 genes were ultimately identified that were always up- or down-regulated regardless of tumor type and the identities of the drivers [209]. The *Gas1* gene (growth arrest-specific 1), which encodes a glycosylphosphatidylinositol (GPI)-linked outer membrane protein that is up-regulated in certain quiescent cell types, was among the most highly down-regulated among these groups. Overriding *Gas1* down-regulation by enforcing its expression during the initiation of BYN HBs markedly slowed the growth of tumors and their derivative cell lines in a manner that required GPI-mediated membrane anchoring of the Gas1 protein [209]. Seventh, regardless of the identities of their genetic drivers, murine HB cell lines should be able to be propagated in vivo as either subcutaneous or pseudo-metastatic tumors in immune-competent host mice of the same genetic background from which they arose. This provides unprecedented opportunities to not only study tumor cell-immune cell interactions in immunologically intact animals but also to examine such questions as to whether these interactions differ with respect to the tumor’s anatomic site. Finally, as already noted, the ability to determine how different drivers impact such properties as metabolism and the ability to trans-differentiate into ECs can be studied under highly controlled in vitro conditions [33].

Unlike the case of ABC-*MYC* tumors, the transformed phenotype of Tet-*MYC* tumors can be reversed simply by re-instating doxycycline and suppressing the expression of the human *MYC* transgene [147,149]. Cell lines from these latter tumors have not been described, but we assume that they should be no more difficult to generate than ABC-*MYC* cell lines. If so, then their transformed state should also be reversible in vitro as well simply by suppressing *MYC* transgene expression with doxycycline. These cell lines would provide a unique opportunity to study both tumor induction and regression under highly controlled and regulated in vitro conditions.

Having enumerated the advantages of murine cell lines, there are of course disadvantages, not the least of which is that they are of murine origin. Also unavoidable is the fact that the driver oncogenes are not under the control of their own promoters and thus would not necessarily be regulated similarly or at the same levels as if they were in their natural context. Because an insufficient number of human HB cell lines have been generated, it is not known whether the means by which they have become immortalized in the process represent the most common pathways and how they relate to the immortalization processes of murine HB cell lines, all of which thus far involve the over-expression of *MYC* or the partial inactivation of *Cdkn2a*. As already mentioned, because such pathways often modulate the response to chemotherapeutic agents, caution is warranted when attempting to extrapolate the findings from drug screening campaigns to actual human tumors although this is an issue when using human HB-derived cell lines as well [191,200,202,203].

## 7. Future Directions

The work discussed above has provided a number of valuable reagents and holds promise for the development of additional ones. Perhaps more importantly, it also points the way to creating the next generation of murine HB cell lines that relies upon recent advances in in vivo Crispr/Cas base editing to introduce patient-derived B, Y, N or other mutations directly into the endogenous loci of mice so as to precisely recapitulate the more human-like nature and regulation of the driver oncogenes [210,211,212].

The finding that at least partial inactivation of the *Cdkn2a* locus is a critical step on the road to in vitro murine HB immortalization suggests that a similar strategy could be used to generate human HB cell lines. At least two approaches to establishing these might be considered. In the first, pre-existing primary tumors could be dissociated into single-cell suspensions, plated and immediately transduced with Crispr/Cas9 vectors such as those we have used that target the *Cdkn2a* locus [32,33]. This method, while attractive, seemingly straight-forward and directly deriving from our own experience, will need to be deployed with the knowledge that primary human cells tend to be much more resistant to immortalization than murine cells and often require the inactivation of multiple TS pathways along with the up-regulation of *TERT* [184,213,214]. The targeting of various combinations of these pathways may thus require substantial optimization in order to identify the most reliable and consistent one(s). Additional steps might include the cultivation of tumor cells under hypoxic conditions, which has been shown to enhance the in vitro immortalization of murine cells but has yet to be shown to benefit human cells [215]. It will also be necessary to ensure that the above approaches do not immortalize cells other than hepatocytes in which cases, cloning or other means of separation may be necessary to select for transformed hepatocytes. Of course, and as already discussed, the use of this approach, while perhaps practical, will likely never yield two cell lines that are precisely alike at the molecular level.

The second potential approach involves the concurrent establishment of genetically defined human HBs and their derivative cell lines. For this, we envision that, after obtaining appropriate IRB approval, human embryonal, fetal or neonatal hepatocytes would first be propagated in immuno-deficient mice that lack the enzyme fumarylacetoacetate hydrolase (*Fah*), and that serve as a model for type 1 hereditary tyrosinemia [216,217,218]. *Fah*−/− mice (and humans) accumulate upstream hepatotoxic intermediates of tyrosine that lead to the gradual death of hepatocytes and eventual hepatic failure. This can be prevented in both mice and humans with the drug nitisinone (also known as NTBC), which blocks the upstream tyrosine catabolic enzyme 4-hydroxyphenylpyruvate dioxygenase and prevents the accumulation of these intermediates. *Fah*−/− mice can also be rescued by the intrasplenic injection of *Fah*+/+ hepatocytes, which gradually replace up to ~70% of the dying *Fah*−/− cells and allow the animals to achieve nitisinone independence. Mice with such “humanized” livers can serve as recipients for HDTVI-mediated delivery of Sleeping Beauty vectors encoding oncoproteins such as B, Y, N, and MYC along with appropriate immortalizing vectors. As discussed in the previous paragraph, Crispr-mediated base editing could be used to introduce oncogenic mutations into endogenous oncogene loci. An alternative approach would involve the genetic manipulation of human hepatocytes in vitro prior to their introduction into the livers of recipient *Fah*−/− mice. Finally, pluripotent human stem cells that have been induced to differentiate into hepatocytes could be substituted for the above primary hepatocytes [219].

Lastly, it is important to mention that established HB cell lines, whether of murine or human origin, provide opportunities to generate derivative cell lines that are resistant to various chemotherapeutic drugs, that are adapted to metastatic growth or that have evolved to escape immune surveillance. Such lines will be likely to yield insights into the means by which these properties are acquired and how they are facilitated or altered by different combinations of oncogenes and TSs.

## 8. Conclusions

The status of human HB-derived cell lines has remained stagnant over the past decade and overwhelmingly monopolized by the highly atypical HepG2 cell line [31]. In contrast, tremendous progress has recently been made in establishing immortalized cell lines from genetically defined murine HBs, with at least 19 of these having been reported and characterized (Table 3) [20,32,33]. Despite expressing four combinations of oncogenic drivers or the single driver *MYC*, these cell lines possess a number of biological and histologic similarities while deregulating a surprisingly similar array of downstream targets that closely recapitulate the transcriptomes of human tumors. These lines afford a number of advantages including their use to test and compare differential sensitivities to pre-existing or new chemotherapeutic drugs and to identify new susceptibilities. At the same time, the ease with which they can be derived from primary HBs provides the ability to examine inter-group similarities and differences whereas the multiple independent cell lines that can be generated with each set of driver also provides a means of assessing intra-group differences [20,32,33]. The vast majority of cell lines derived from the four B/Y/N tumor groups are also tumorigenic and can generate pseudo-metastases. This allows different tumor groups to be propagated in vivo in the immuno-competent mice from which they were derived while permitting studies of tumor-immune system interactions that were heretofore impossible to perform with human HB cell lines. Finally, the unexpected finding that BN cell lines can reversibly acquire EC-like properties in response to short-term hypoxia provide a novel and readily quantifiable means by which to study the biochemical and molecular basis for this epithelial-to-mesenchymal transition, the role it plays in promoting tumor growth and the generation of the tumor neovasculature. Overall, the ideas gained from these studies suggest novel ways by which genetically defined human HB cell lines can be generated in future work.

## Figures and Tables

**Table 1:** Summary of human HB cell lines.

Name of Cell Line	Patient Origin	Relevant Mutations, De-Regulated Oncogenes and/or Tumor Suppressors	Comments	Reference(s)
HepG2	15 years male	β-cat (in-frame deletion), *TERT* promoter^G222A^		[34,35,36,37,38]
Huh6	1 years male	β-catenin^G34V^		[37,39,40,41]
HepT1	3 years female			[42]
HepT3	9 months male			[43]
Hep293TT	5 years female	β-catenin (in-frame deletion)		[44]
HepU1, HepU2	3 years male		2 lines from the same HB	[45]
HB1	6 months male	High MYC & H-RAS expression		[46]
OHR	4 months male	TP53, β-catenin^R281H^		[47]

**Table 3:** Summary of murine hepatoblastoma cell lines.

Name of Cell Line	Relevant Mutations, De-Regulated Oncogenes, and/or Tumor Suppressors	Comments	Reference(s)
MHB-2 and others	*Hras*, *Braf*, *Egfr* ~20%: *Ctnnb1 & Apc* mutations in older mice	DEN/PB-treated mouse ^1^, DEN-induced only resemble HCCs, non-tumorigenic	[61,135,136]
BY 1–5, BY 21	*CTNNB1*^del90^, *YAP*^S127A^	Immortalized via Crispr mutagenesis of *Cdkn2a*	[32]
BYN 1–3	*CTNNB1*^del90^, *YAP*^S127A^, *NRF2*^L30P^	Immortalized via Crispr mutagenesis of *Cdkn2a*	[32]
BN 1,2	CTNNB1^del90^, NRF2^L30P^	Immortalized via Crispr mutagenesis of *Cdkn2a*	[33]
YN 1–3	*YAP*^S127A^, *NRF2*^L30P^	Immortalized via Crispr mutagenesis of *Cdkn2a*	[33]
NEJF1,2,3,4,5,6,10	ABC-*MYC*		[20]

^1^ DEN = diethylnitrosamine, PB = phenobarbital.

**Table 4:** Advantages and Disadvantages of current human and murine HB cell lines.

Species	Advantages	Disadvantages
Human	Human in origin; behaviors will therefore be human specific All primary and secondary mutations and epigenetic changes should be present Mutant drivers are in their natural context	Very few lines available and are poorly characterized May not retain the biological properties of the primary tumor, especially if propagated for long periods Certain clones may be selected during in vitro establishment No control possible over the molecular drivers Cannot be propagated in immune-competent mice Difficult to ascertain the role of individual drivers Unlikely that any two cell lines will be precisely alike Derived from genetically heterogeneous individuals
Mouse	Generation is easy and efficient Can be maintained in the same standard growth media Genetically defined Isogenic backgrounds Theoretically possible to obtain bespoke HBs and cell lines driven by a combination of oncogene/tumor suppressors Different variations of the same drivers can be studied Opportunities to identify common genes and pathways involved in transformation (Gas1) Can be propagated in vivo in immune-competent hosts Multiple cell lines can be generated to confirm specificity and consistency of various properties and behaviors Most are metastatic from the onset Some can trans-differentiate into endothelial cells Transformation should be reversible	Not human Driver genes are not in their natural context and are thus not subject to their normal control Genes leading to immortalization (e.g., *MYC* and *Cdk2na*) likely alter chemotherapeutic sensitivities Do not deregulate other, minor oncogenes and/or tumor-suppressors Primary tumors are not metastatic

## Data Availability

The original contributions presented in this study are included in the article/Supplementary Material. Further inquiries can be directed to the corresponding author.

## Supplementary Materials

The following supporting information can be downloaded at: https://www.mdpi.com/article/10.3390/cells14242013/s1, File S1: List of genes that were significantly more down-regulated in ABC-*MYC* HBs versus Tet-*MYC* HBs as indicated in Supplementary File S2, Figure S1A; File S2: Figure S1. Transcriptomic comparisons among primary murine HBs generated by Tet-*MYC*, ABC-*MYC* and B/Y/N combinations. (A). Heatmap depicting log_2_-fold differences versus normal control livers using hierarchical clustering (hclust). Although all six of the indicated HB groups are closely related, the Tet-*MYC/*ABC-*MYC* tumor groups are distinct from each of the four B/Y/N groups. ABC-*MYC* HBs also differ from all others by virtue of their more pronounced down-regulation of ~400 genes involved in steroid and glycogen metabolism (red box and Supplementary File S1). (B). Expression of *Prkdc* transcripts among each of the groups shown in A. ***: *p* < 0.01; ****: *p* < 0.001. Figure S2. Up-regulation of endogenous β-catenin (CNTTB1), YAP and NRF2 in Tet-*MYC* livers and HCCs. Cytoplasmic (C) and nuclear (N) fractions from Tet-*MYC* livers (L) and tumors (T) were resolved by SDS-PAGE and subjected to immuno-blotting for the indicated proteins. Histone H3 (H3) and GAPDH were used as controls for protein loading and to assess nuclear and cytoplasmic localization, respectively.

## Abbreviations

The following abbreviations are used in this manuscript:

B	A patient derived mutant form of the β-catenin/CTNNB1 TF, especially CTNNB1^del90^
BN	Tumor or cell line driven by the combination of B and N oncoproteins
BY	Tumor or cell line driven by the combination of B and Y oncoproteins
BYN	Tumor or cell line driven by the combination of B and Y, and N oncoproteins
DEN	Diethylnitrosamine
EC	Endothelial cell
Fah	Fumarylacetoacetate hydrolase
HB	Hepatoblastoma
HCC	Hepatocellular carcinoma
N	A patient-derived constitutively active form of the NRF2 transcription factor
PB	Phenobarbital
TF	Transcription factor
TS	Tumor suppressor
Y	A constitutively activated form of the yes-associated protein transcription factor (YAP^S127A^)
YN	Tumor or cell line driven by the combination of Y and N oncoproteins
