# Supplementary material for: Hepatoblastoma Cell Lines: Past, Present and Future
Source: Cells. 2025 Dec 17;14(24):2013. doi: 10.3390/cells14242013 (PMC12731888; doi:10.3390/cells14242013)
Supplement: Supplementary file 1 [file cells-14-02013-s001.zip › SupplementaryFileS2.pdf]

SUPPLEMENTARY MATERIAL

Materials and Methods

Computational work.

All RNA-seq raw data were downloaded from the GEO database under accession numbers GSE157623 (BYN dataset), GSE193124 (ABC MYC dataset) and GSE303515 (Tet-MYC dataset). Raw FASTQ files were processed using the nf-core/rnaseq v3.12.0 pipeline. Reads were aligned to the GRCm38 reference genome (annotation: Ensembl release 86 .gtf). Raw read counts were used as input for differential expression analysis with the DESeq2 R package (version 1.42.1). Log_2 fold-change values relative to normal liver samples were used to generate heatmaps. using the ComplexHeatmap R package. To enable cross-dataset comparisons of Prkdc transcripts, mean expression levels in normal livers were manually set to 1 for each dataset. Tumor sample expression values were then normalized and expressed relative to liver expression.

Tumor cell fractionation and immunoblotting.

All procedures have been described previously and were performed on samples of tumors or control livers that had previously been flash frozen in liquid nitrogen and then maintained at -80C [25]. Nuclear and cytoplasmic fractions were isolated using a Subcellular Protein Fractionation Kit for Tissues according to the directions provided by the vendor (Thermo Fisher, Inc., Waltham, MA). After quantification of each fraction, 10 µg of each sample was subjected to SDS-PAGE and western blotting using PVDF membranes (Thermo Fisher) [25,26]. The following antibodies were used for the detection of proteins of interest (β-catenin: Abcam, Cambridge, UK, cat. no. ab16051, [1:4000]; YAP: Cell Signaling Technology, Danvers, MA, cat. no. 4912, [1:500]; NRF2: Sigma-Alddrich, St. Louis, MO, cat. no. SAB2701989, [1:500]; Histone H3: Cell Signaling Technology, cat. no. 9715, [1:1000]; GAPDH: Sigma-Aldrich, cat. no. G8795, [1:10,000]. Immunoblots were developed using a Thermo Scientific Pierce ECL Western Blotting Substrate Kit (Thermo Fisher, Waltham, MA).

Statistical analyses.

To enable cross-platform comparisons in Fig. 1B, the mean expression levels of control liver samples were manually set to 1 in each dataset. Tumor values were then normalized and expressed relative to liver expression within each group. Statistical analysis was performed using GraphPad Prism v10.5.0. An ordinary one-way ANOVA was applied to compare expression values across all groups, assuming a Gaussian distribution. Post-hoc comparisons were conducted using Fisher's Least Significant Difference (LSD) test.

Supplementary Figures

A B

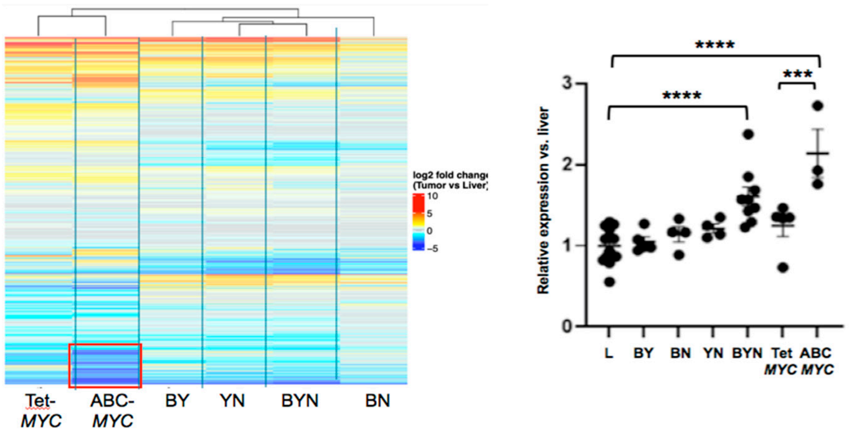

Supplementary Figure S1. Transcriptomic comparisons among primary murine HBs generated by Tet-*MYC*, ABC-*MYC* and B/Y/N combinations. (A). Heatmap depicting $\log_2$-fold differences versus normal control livers using hierarchical clustering (hclust). Although all six of the indicated HB groups are closely related, the Tet-*MYC*/ABC-*MYC* tumor groups are distinct from each of the four B/Y/N groups. ABC-*MYC* HBs also differ from all others by virtue of their more pronounced down-regulation of ~400 genes involved in steroid and glycogen metabolism (red box and Supplementary File S1). (B). Expression of *Prkdc* transcripts among each of the groups shown in A. ***: $p < 0.01$; ****: $p < 0.001$.

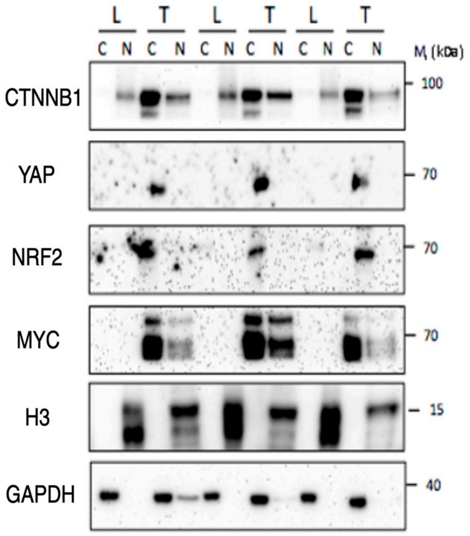

Supplementary Figure S2. Up-regulation of endogenous β-catenin (CNTTB1), YAP and NRF2 in Tet-*MYC* livers and HCCs. Cytoplasmic (C) and nuclear (N) fractions from Tet-*MYC* livers (L) and tumors (T) were resolved by SDS-PAGE and subjected to immuno-blotting for the indicated proteins. Histone H3 (H3) and GAPDH were used as controls for protein loading and to assess nuclear and cytoplasmic localization, respectively.
